# Segregated expressions of autism risk genes *Cdh11* and *Cdh9* in autism-relevant regions of developing cerebellum

**DOI:** 10.1186/s13041-019-0461-4

**Published:** 2019-05-02

**Authors:** Chunlei Wang, Yi-Hsuan Pan, Yue Wang, Gene Blatt, Xiao-Bing Yuan

**Affiliations:** 1 Hussman Institute for Autism, Baltimore, MD 21201 USA; 2 Key Laboratory of Brain Functional Genomics (Ministry of Education and Shanghai), Institute of Brain Functional Genomics, School of Life Science and the Collaborative Innovation Center for Brain Science, East China Normal University, Shanghai, 200062 People’s Republic of China; 3 Department of Anatomy and Neurobiology, University of Maryland School of Medicine, Baltimore, MD 21201 USA

**Keywords:** ASD, Cdh11, Cdh9, Cerebellum, Development, In situ hybridization

## Abstract

Results of recent genome-wide association studies (GWAS) and whole genome sequencing (WGS) highlighted type II cadherins as risk genes for autism spectrum disorders (ASD). To determine whether these cadherins may be linked to the morphogenesis of ASD-relevant brain regions, in situ hybridization (ISH) experiments were carried out to examine the mRNA expression profiles of two ASD-associated cadherins, Cdh9 and Cdh11, in the developing cerebellum. During the first postnatal week, both Cdh9 and Cdh11 were expressed at high levels in segregated sub-populations of Purkinje cells in the cerebellum, and the expression of both genes was declined as development proceeded. Developmental expression of Cdh11 was largely confined to dorsal lobules (lobules VI/VII) of the vermis as well as the lateral hemisphere area equivalent to the Crus I and Crus II areas in human brains, areas known to mediate high order cognitive functions in adults. Moreover, in lobules VI/VII of the vermis, Cdh9 and Cdh11 were expressed in a complementary pattern with the Cdh11-expressing areas flanked by Cdh9-expressing areas. Interestingly, the high level of Cdh11 expression in the central domain of lobules VI/VII was correlated with a low level of expression of the Purkinje cell marker calbindin, coinciding with a delayed maturation of Purkinje cells in the same area. These findings suggest that these two ASD-associated cadherins may exert distinct but coordinated functions to regulate the wiring of ASD-relevant circuits in the cerebellum.

**Electronic supplementary material:**

The online version of this article (10.1186/s13041-019-0461-4) contains supplementary material, which is available to authorized users.

## Background

Autism spectrum disorder (ASD) is a neurodevelopmental condition characterized by compromised social interaction and communication abilities, and by repetitive/stereotyped behaviors. It is generally believed that pathological changes in the brain that contribute to ASD occur during early developmental stages and have long-lasting effects on the brain and behavior [1]. Genetic factors have been shown to play a pivotal role in the etiology of ASD, although environmental risk factors also contribute significantly to the prevalence of ASD [2, 3]. In recent years, rapid progress has been made in the identification of genes whose mutations significantly increase the susceptibility of ASD of ASD. The next challenge for autism research is to clarify how these genetic variants are linked to core symptoms of ASD [4–6].

An important strategy for understanding how risk genes are linked to ASD etiology is to analyze whether these genes are expressed in brain regions directly relevant to the neurobiology and pathology of ASD during a critical period of brain development. Emerging evidence shows that deficits in the structure and function of the cerebellum (CB) are closely related to the pathology of ASD [7–12]. ASD risk genes have been shown to exhibit convergent expression in the developing CB [13]. In addition to its well-established function in motor control, the CB is increasingly recognized for its role in non-motor functions, including language, cognition, emotion, affection, and social interaction [12]. It is believed that CB plays an essential role in the formation of basic social capabilities during postnatal development [7–12]. Extensive clinical observations support the idea that CB abnormalities are linked to behavioral characteristics of ASD, including difficulties in the initiation and completion of motor activities and non-motor functions such as social interaction and language [7, 14]. The CB is one of the most vulnerable structures in ASD, and the most consistent pathological changes in the brain of individuals with ASD occur in the CB, including CB hypoplasia and decreased numbers of Purkinje cells (PCs). These changes are found in areas that control cognitive and language functions, i.e., Crus I and Crus II areas of the lateral hemisphere and the posterior vermis [15]. Consistent with pathological changes in the CB, individuals with ASD frequently exhibit deficits in motor control in addition to core ASD symptoms of difficulties in language and social interaction. Clinical observations also showed that lesions or injury of the CB in early childhood lead to autitic-like behavioral changes at a very high frequency [16]. Early-life cerebellar injury is considered as the strongest exogenous risk factor of ASD [16–18]. Animal studies have confirmed that an early-life cerebellar lesion leads to deficits in social behavior and vocalization [19, 20]. Conditional knockout of ASD risk genes in PCs is sufficient to cause autistic-like behavioral changes [8, 9]. Collectively, these findings indicate the importance of the CB in the development of ASD.

Cadherin family members are a large group of cell adhesion molecules that play important roles in the wiring of brain circuits by mediating homophilic and heterophilic cell-to-cell interactions [21]. Recent genetic findings have implicated type II cadherins as risk genes for ASD [22–25]. How these cadherins could be associated with ASD is largely unknown. Although several studies have been carried out to describe the expression of several type II cadherins in different areas of developing brains [26–42], the expression of cadherins in autism-relevant structures have not been carefully analyzed. In this study, we investigated two recently identified ASD risk genes that code for type II cadherins, Cdh9 and Cdh11. These two genes exhibited a unique complementary expression pattern in ASD-relevant areas of the CB in neonatal mice, including the dorsal vermis and the lateral hemisphere. The expression of Cdh11, but not that of Cdh9, was correlated with a delayed maturation of PCs in dorsal vermis. These findings imply that genetic variants of these two ASD risk genes may contribute to ASD-relevant motor and/or non-motor functions mediated by cerebellar circuits.

## Methods

### Animals

All procedures involving animals were carried out in accordance with the NIH Guide for the Care and Use of Laboratory Animals and were approved by the Institutional Animal Care and Use Committee of the University of Maryland School of Medicine (protocol number #0515017) and the Hussman Institute for Autism (protocol number #06012015D). Wild type C57/B6 mice were obtained from the veterinary facility of the University of Maryland School of Medicine. The day of birth was designated as postnatal day 0 (P0).

### Paraformaldehyde fixation and preparation of brain sections

Postnatal mice were anesthetized by intraperitoneal (IP) administration of Ketamine (Zetamine, NDC 13985–702-10, VETone) at a dose of 100 mg per kg of body weight and then transcardially perfused with 4% paraformaldehyde (PFA). Brains were dissected and post-fixed with 4% PFA overnight. Sagittal or coronal sections (25 μm for ages P0 – P10, 20 *μ*m for age P30) were made with a cryostat, mounted on slides (Fisherbrand, 12–545-C), air dried, and stored at − 80 °C until used.

### Preparation of RNA probes and in situ hybridization

In situ hybridization was carried out as previously described with minor modifications [43]. Mouse RNAs were obtained from a P7 mouse brain. RT-PCR was performed to obtain probe templates (according to the sequences obtained from the Allen Developing Mouse Brain Atlas) for Cdh9 (Entrez gene ID: 12565) and Cdh11 (Entrez gene ID: 12552) genes. Primers for Cdh9 probe template were forward: TGA AAT GTC TGG AGT TGG TAC G and reverse: ATA TGC TGT GAC TTG TCC GAT G. Primers for Cdh11 probe template were forward: AAG TCC CAG TGG CCA TCA and reverse: TGT CGT GGC AGA CTC CAA. The resulting PCR products were cloned into the T-easy plasmid (#A1360, Promega). The recombinant plasmids thus generated were linearized, and in vitro transcription was performed to produce both sense and antisense RNA probes using a DIG (digoxigenin) RNA labeling kit (#11175025910, Roche).

For in situ hybridization, sections were dried at 50 °C for 15 min, re-fixed in 4% PFA for 15 min, washed with 1x PBS for 10 min, digested with 0.5 mg/mL Proteinase K (#EO0491, Thermo Scientific) for 3 min, fixed again with 4% PFA for 20 min, and then rinsed in 1x PBS for 10 min. After prehybridization for 2 h at 65 °C in hybridization buffer [50% formamide, 5x saline-sodium citrate buffer (SSC, pH 4.5), 5 mM EDTA, 1 mg/mL 3-[(3-cholamidopropyl) dimethylammonio]-1-propanesulfonate (CHAPS), 1x Denhardt’s solution, 200 μg/mL heparin, 0.1% Tween-20, 500 μg/mL yeast tRNA], the sections were hybridized overnight with 1–3 μg/mL of DIG-labeled probe at 65 °C. Hybridized sections were washed at 65 °C with 2x SSC for 5 min and then with a buffer containing 50% formamide and 2x SSC (pH 4.5) for 90 min. Following another wash at room temperature for 20 min with KTBT solution (50 mM Tris-HCl (pH 7.5), 0.15 M NaCl, 10 mM KCl, and 0.5% Tween-20), sections were blocked in a solution containing 20% fetal calf serum in KTBT for 2 h and then incubated overnight at 4 °C in the blocking solution containing an alkaline-phosphatase-conjugated anti-DIG antibody (1:1000; Roche). The color reaction was performed after washing with KTBT solution with 0.35 mg/mL nitrotetrazolium blue chloride (NBTC) and 0.175 mg/mL 5-bromo-4-chloro-3-indoxyl phosphate, p-toluidine salt (BCIP) at room temperature for 2 h.

### Immunohistochemistry

Immunohistochemistry was performed on sections after in situ hybridization. Briefly, sections were incubated with the primary antibody solution (0.3% Triton, 1% BSA, 1x PBS) containing anti-calbindin polyclonal antibody (rabbit, 1:500; Sigma) overnight at 4 °C. Sections were then washed with 1x PBS for 15 min at room temperature followed by incubation with the secondary antibody (Rhodamine Red donkey anti-rabbit IgG, Jackson ImmunoResearch Inc.) (1:500 in 1x PBS) for 2 h. Sections were washed with 1x PBS and covered in mounting medium (Fluoroshield, F6182, Sigma).

### Microscopy and image processing

Bright field and fluorescent images were taken with a Zeiss Axio Zoom V16 Stereo Microscope. Some colored images were converted to black and white.

## Results

### Segregated expression of Cdh11 and Cdh9 in the cerebellum

The expression of Cdh11 and Cdh9 in developing mouse brain was demonstrated by in situ hybridization. The specificity of probes was tested by comparing hybridization signals from sense and antisense probes on two adjacent sagittal sections at P0 (Additional file 1: Figure S1). Using the antisense probes, we found that both genes were expressed in the CB at high levels during the first postnatal week, consistent with results of the Allen Developing Mouse Brain Atlas. Cdh9 mRNAs were primarily detected in the intermediate part of the CB (the paravermis), whereas Cdh11 mRNAs were observed in more lateral areas (hemisphere) and in a small region of the dorsal part of the vermis.

**Fig. 1:**
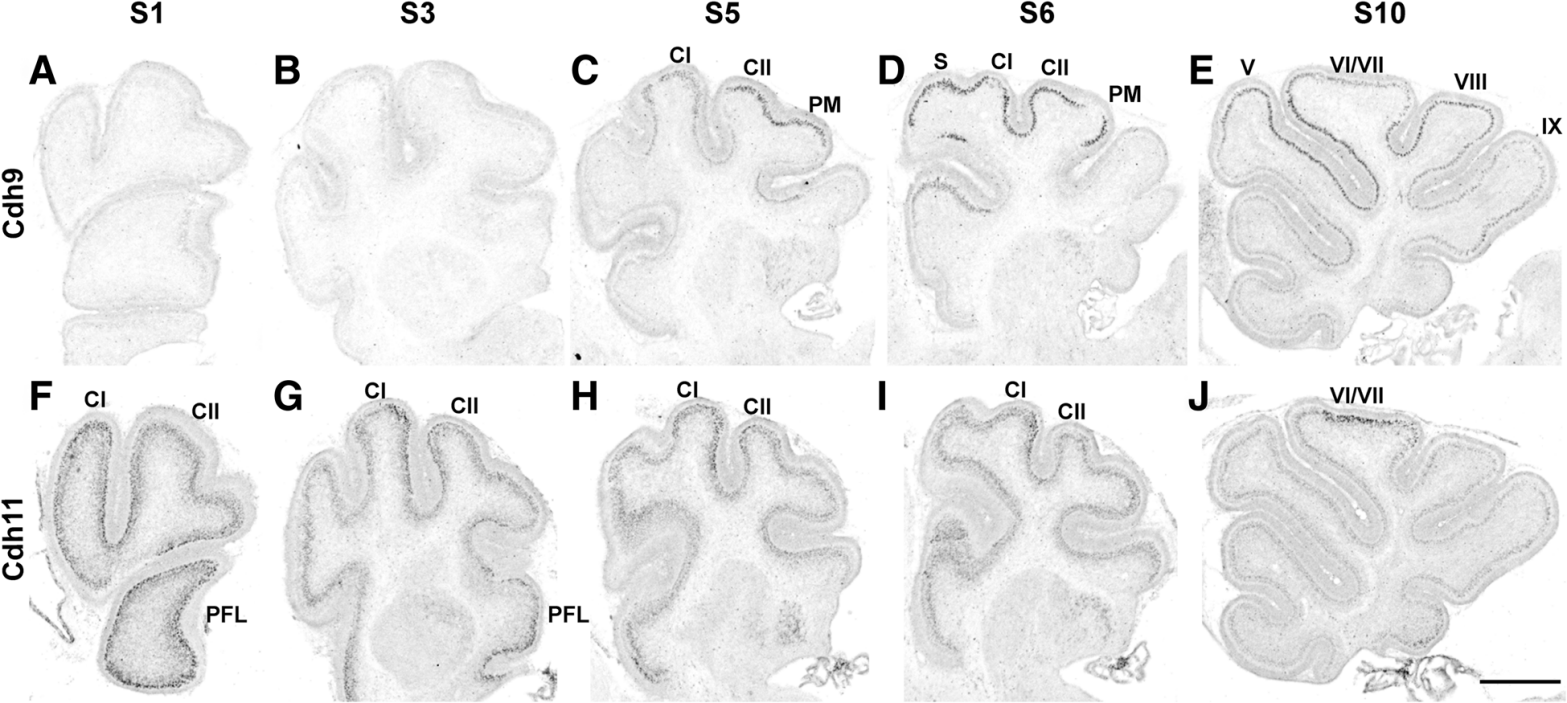
Expressions of Cdh9 and Cdh11 in neonatal cerebellum. In situ hybridization of Cdh9 and Cdh11 mRNAs was performed on serial sagittal sections of two different brains at P4. Sagittal levels of the cerebellum are defined as follows: S0 is the most lateral sagittal level, and S10 is the most medial section of the cerebellum. **a**-**e** sections show Cdh9 expression signals and **f-j** sections show Cdh11 expression signals at different sagittal levels. In general, Cdh9 is expressed in more medial levels and in multiple lobules, whereas Cdh11 is highly expressed in most lateral levels, including Crus I (CI) and Crus II (CII), and its expression is restricted to a narrow band in lobules VI/VII of the vermis. These data show that Cdh9 and Cdh11 are expressed in the Purkinje cell layer in different domains of the cerebellum. C1: Crus I; CII: Crus II; S: Simple lobule; PM: Paramedian lobule; PFL: Paraflocculus. Scale: 500 μm

To better characterize the expression pattern of Cdh9 and Cdh11 in the CB, in situ hybridization was carried out on serial sagittal sections for each of these two genes. As shown in the Fig. 1, in sagittal sections of the vermis of postnatal day 4 (P4) mice, Cdh9 mRNAs were detected in the PC layer in several lobules, including lobules V, VI/VII, VIII, IX, and X. In contrast, at a similar sagittal level, strong Cdh11 expression appeared to be mainly restricted to the central part of lobules VI/VII (Fig. 1j). Interestingly, Cdh9 exhibited a much lower expression level in the central part of lobules VI/VII compared to surrounding areas (Fig. 1e). In the medial part of the CB, Cdh9 was expressed at a high level in multiple lobules including the Simple lobule (LS), Crus I, Crus II, and the Paramedian lobule (Fig. 1c, d). However, Cdh11 signal appeared to be confined mostly to the central part of the Crus I area (Fig. 1h, i). In the more lateral part of cerebellar hemispheres, Cdh9 mRNAs were not detected (Fig. 1a, b), whereas Cdh11 mRNAs were abundant in Crus I and Crus II areas and some ventral lobules (Fig. 1f, g). These data showed that these two cadherins are expressed in a segregated manner.

### Segregated expression of Cdh9 and Cdh11 in ASD-related regions

**Fig. 2:**
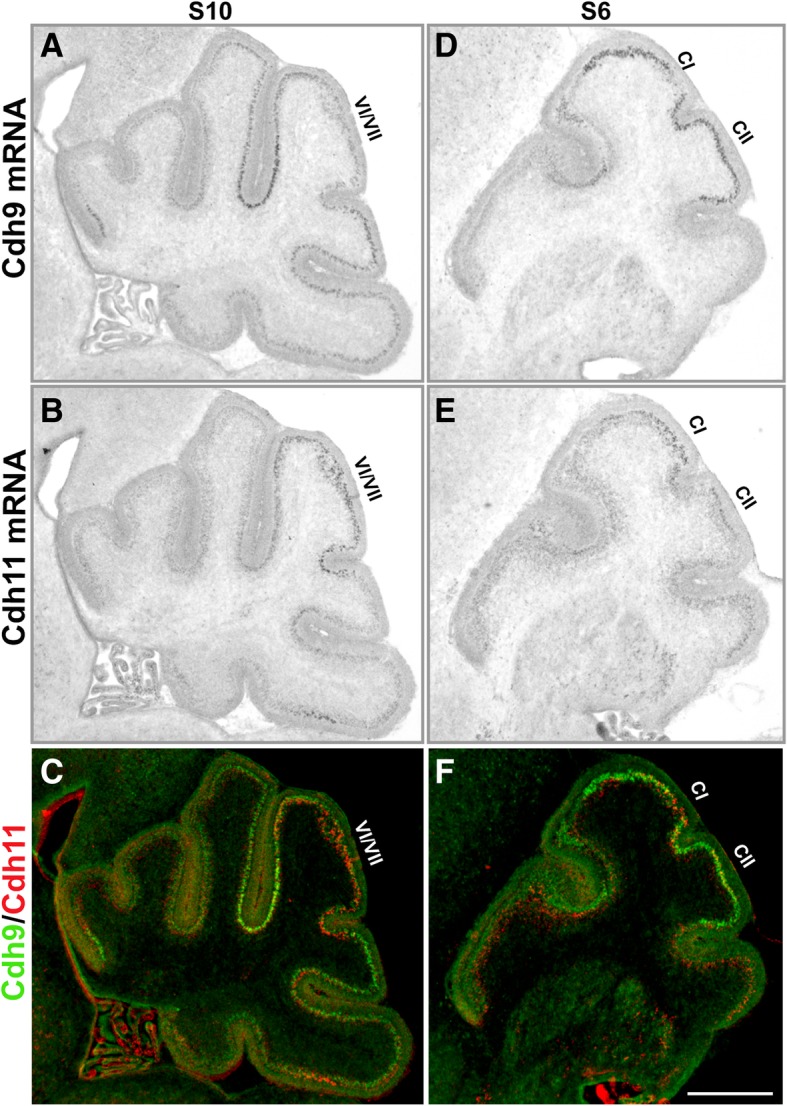
Segregated expressions of Cdh9 and Cdh11 in ASD-associated cerebellar areas. In situ hybridization of Cdh9 and Cdh11 were performed on adjacent sagittal sections (P4) at different levels. **a-c** sections show in situ hybridization signals at level S10, and **d-f** sections show expression signals at level S6. Images were merged in (**c**) and (**f**) with signal intensity coded with pseudo colors. In lobules VI/VII of vermis, an ASD-associated area of the cerebellum, Cdh11 was expressed in the central part and Cdh9 was expressed in the surrounding areas, forming a segregated expression pattern. In Crus I (CI) and Crus II (CII) of the hemisphere, another ASD-associated area of the cerebellum, they formed a similar segregated expression pattern with Cdh11 in the central part of Crus I and Cdh9 in the surrounding areas. Scale bar: 500 μm

In situ hybridization on adjacent sagittal sections was then performed to compare the expression levels of these two cadherins in local areas related to the ASD, including lobules VI/VII of the vermis and the Crus I and Crus II areas of the lateral hemisphere. As shown in Fig. 2, in both lobules VI/VII and Crus I and Crus II areas, Cdh9 and Cdh11 exhibited segregated and complementary expression patterns, with high level of expression of Cdh11 in the central domain and Cdh9 in the flanking areas, suggesting that these two genes may coordinate the wiring of local circuitries in these ASD-related areas during early postnatal development.

### Low level of Cdh11 expression in Bergmann glial cells in the cerebellum

**Fig. 3:**
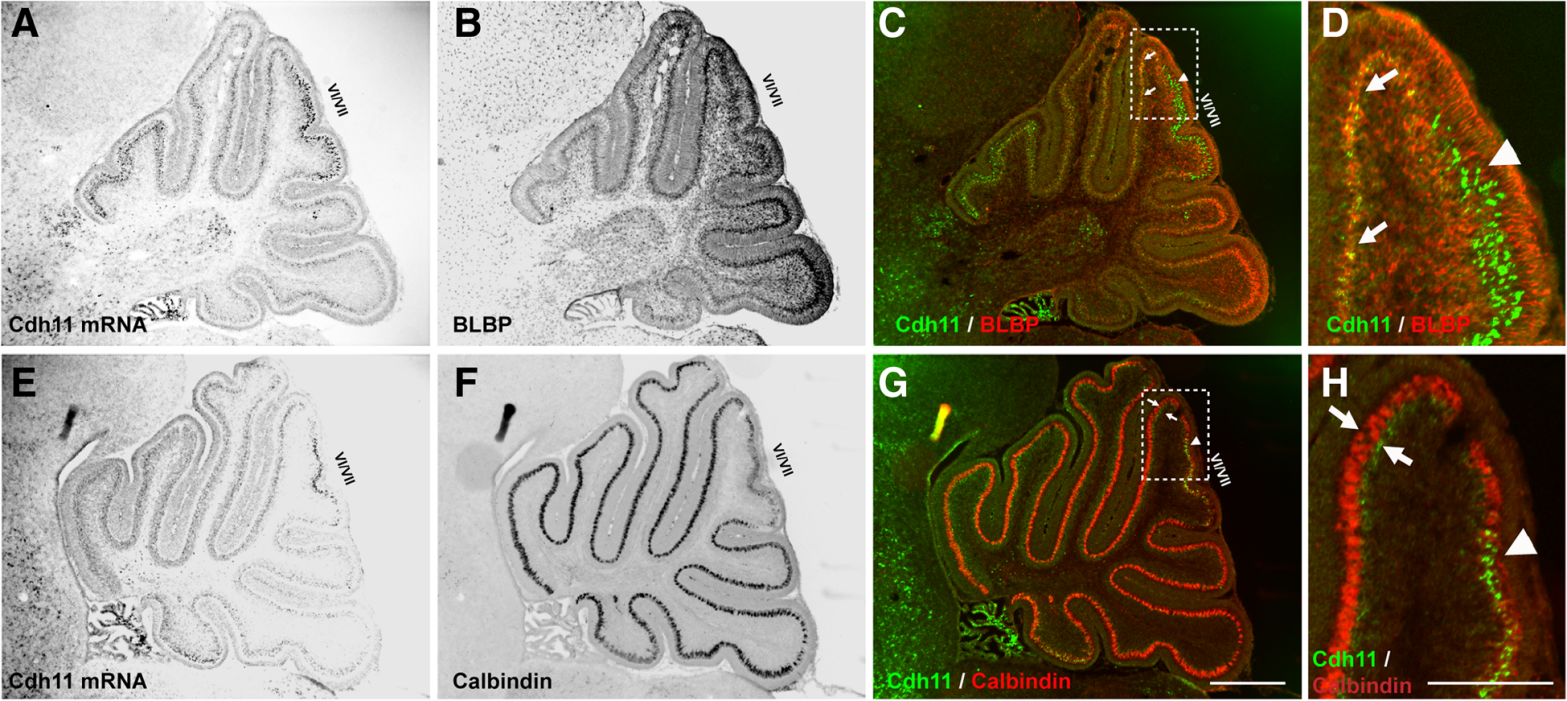
Low level of expression of Cdh11 in Burgmann glial cells. In situ hybridization of Cdh11 followed by calbindin or BLBP staining was performed on P4 sagittal sections. **a-d** Colocalization of BLBP signal (red in (**c**) and (**d**)) with Cdh11 in situ hybridization signal (green in (**c**) and (**d**)) in areas except the central region of lobules VI/VII is demonstrated. **d** The enlarged image of the boxed area in **(c)** shows the weak Cdh11 expression signal in BLBP-positive Burgmann glial cells. In the central area of lobules VI/VII, strong Cdh11 expression is not colocalized with BLBP signal (**c** and **d**, arrow heads). **e-h** a weak Cdh11 signal (green in (**g**) and (**h**)) is present underneath (inferior to) the Purkinje cell layer as indicated by calbindin signal (red in (**g**) and (**h**), arrows). Strong Cdh11 expression signal is within the calbindin-positive cell layer. Scale bar for (**a-c**) and (**e-g**): 500 μm. Scale bar for (**d**) and (**h**): 200 μm

In addition to the high expression of Cdh11 in ASD-relevant areas, a low level of Cdh11 expression signal was detected in areas very close to the PC layer across the whole CB in early postnatal mice (P4). To determine what cells exhibit this low level of Cdh11 signal, brain sections were stained with the antibody against calbindin, the specific marker of PCs, or with the antibody against brain lipid-binding protein (BLBP), a marker for Bergmann glia in the CB, following in situ hybridization of Cdh11. Results showed weak Cdh11 hybridization signal overlapped with BLBP staining below the PC layer (Fig. 3g, h), suggesting that Cdh11 is expresses at a low level in Bergmann glial somata in neonatal mice (Fig. 3c, d). High level of Cdh11 expression was seen in the central area of lobules VI/VII, above the Bergmann glial somata layer (Fig. 3c, d) but within the calbindin-positive PC layer (Fig. 3g, h).

### Cdh11 expression in Purkinje cells in adulthood

**Fig. 4:**
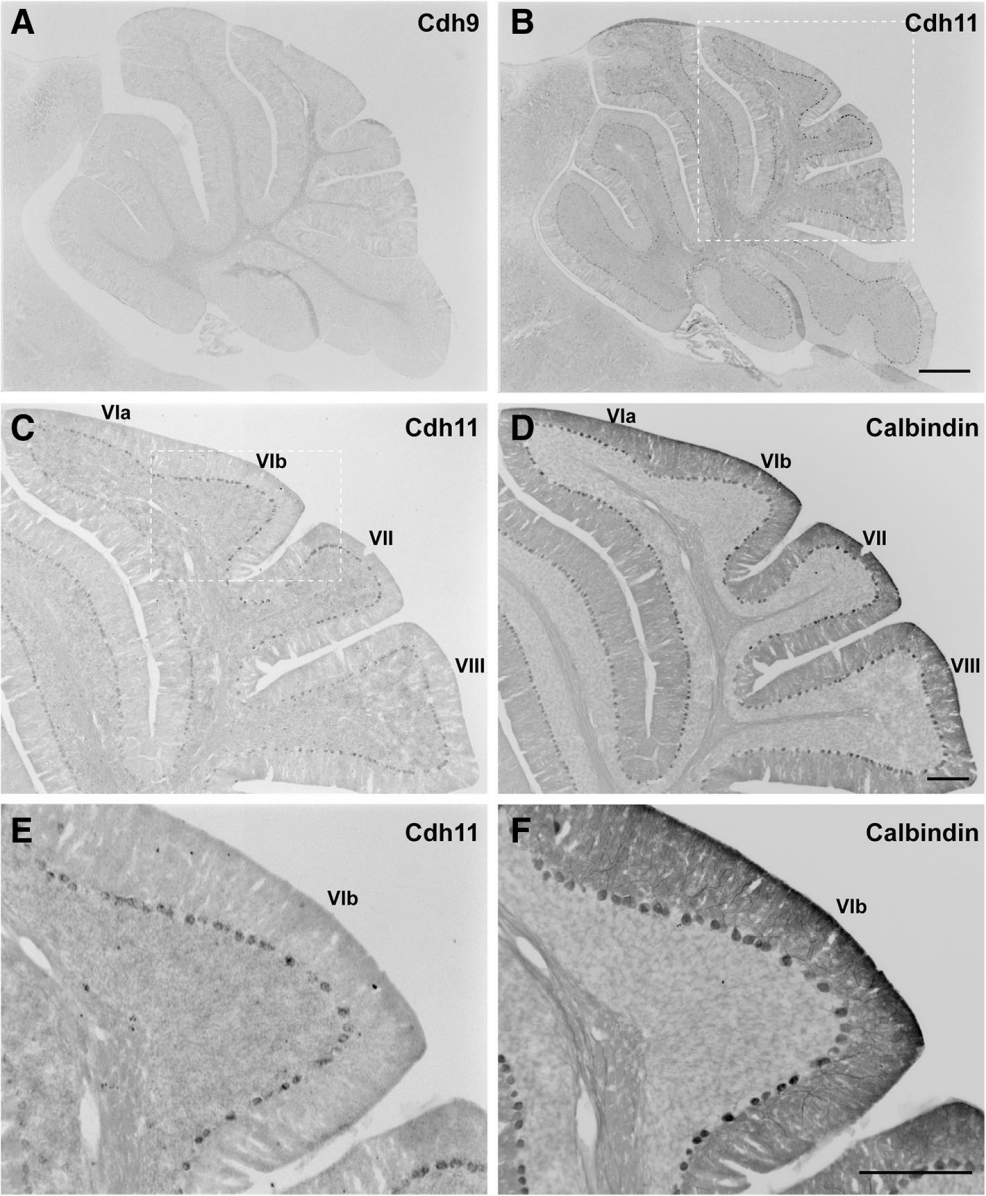
Expressions of Cdh9 and Cdh11 in young adult mice. In situ hybridization was performed on sagittal sections of P30 mouse cerebella. **a** In situ hybridization of Cdh9 shows a lack of expression throughout the cerebellum. **b** In situ hybridization of Cdh11 shows low level of expression in all lobules of the cerebellum. **c**, **d** Enlarged images of boxed area in (**b**) show in situ hybridization signal (**c**) and counterstained calbindin signal (**d**). **e**, **f** Enlarged images of boxed area in (**c**) show in situ hybridization signals (**e**) and counterstained calbindin signal (**f**), indicating that Cdh11 is expressed in Purkinje cells. Scale bars: 500 μm in (**a**, **b**) and 200 μm in (**c**, **d**, **e**, and **f**)

After the first postnatal week, in situ hybridization signals for both Cdh9 and Cdh11 in the specific cerebellar areas declined. At the young adult stage (P30), Cdh9 expression was completely absent in the CB, whereas Cdh11 was expressed at a low level (Fig. 4a, b). At this stage, Cdh11 mRNAs were seen in the entire CB, although its expression in the central area of lobules VI/VII was slightly higher (Fig. 4b). Double staining with the PC marker calbindin showed that Cdh11-positive cells were PCs (Fig. 4c-f), consistent with the previous finding that Cdh11 is expressed in PCs in the adult mouse cerebellum [37]. These data suggest that Cdh11 may play a long-term role in regulating the function of CB circuits and that Cdh9 is mainly involved in the early developmental processes of the CB.

### Correlation of Cdh9 and Cdh11 expressions with Calbindin expression

**Fig. 5:**
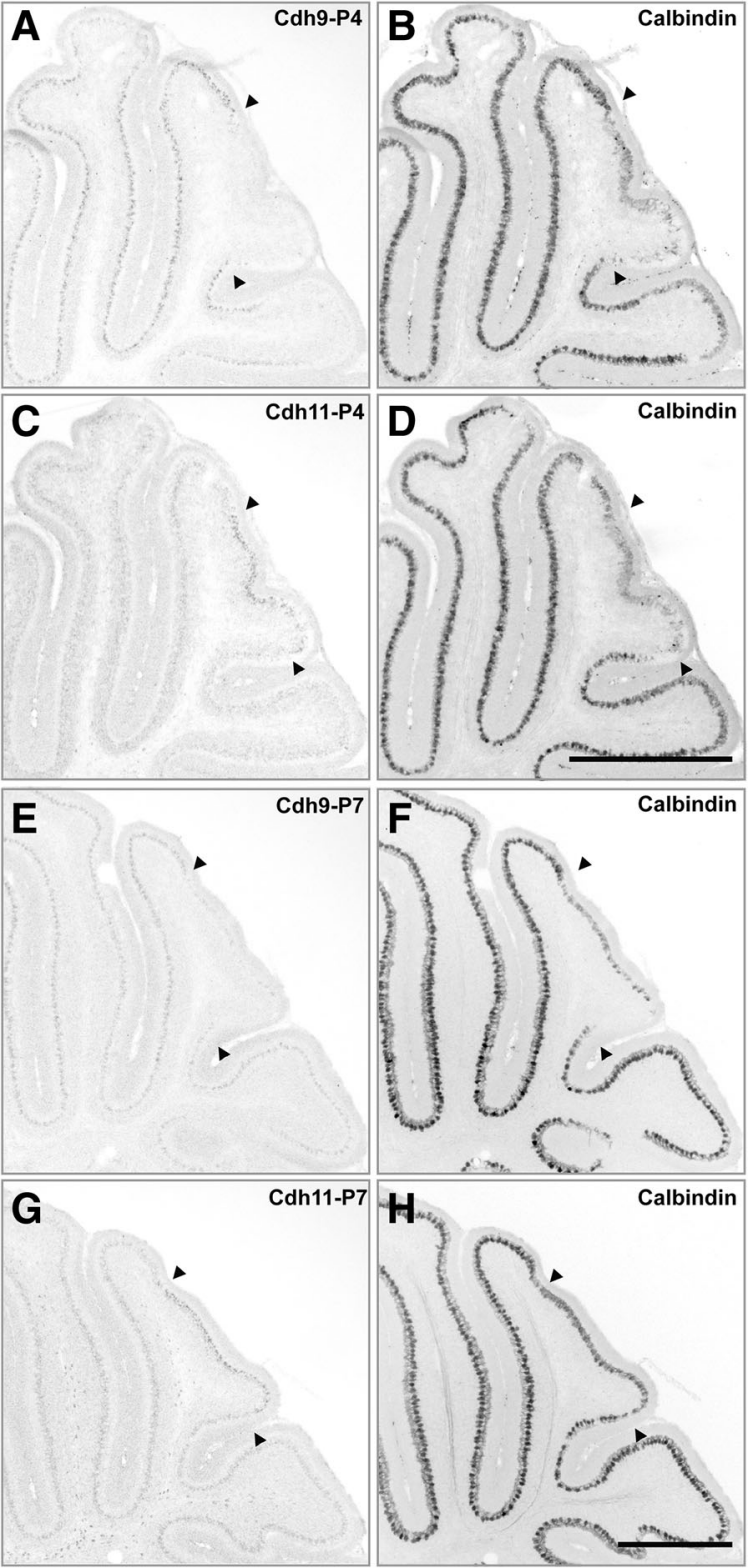
Relationship between cadherins and calbindin during the first postnatal week. In situ hybridization followed by calbindin staining was performed on P4 (**a-d**) and P7 (**e-h**) sagittal sections. **a** and **b** sections show Cdh9 expression signals in high calbindin-expressing areas. **c** and **d** sections show Cdh11 in low calbindin-expressing areas (central area of lobules VI/VII). A similar expression pattern was observed at stage P7 (**e-h**). Arrow heads mark the area of central VI/VII. Scale bars: 500 μm

During the first postnatal week, PCs complete their migration to the final position, form the PC layer, and start their dendritic development [44]. The calcium buffering protein calbindin is known to be expressed in developing PCs, and calbindin expression levels are correlated with the maturation status of PCs [45]. In situ hybridization was performed for each of the two ASD-associated cadherin genes followed by immunostaining of the same section with the calbindin antibody. Calbindin expression was high in Cdh9-expressing regions but was very low in the region with high Cdh11 expression in lobules VI/VII of the vermis at P4 (Fig. 5a-d). An overall parallel correlation between Cdh9 and calbindin expression and an overall inverse correlation between Cdh11 and calbindin expression were observed in the same areas at P7 (Fig. 5e-h). The low calbindin expression in Cdh11-expressing neurons suggests that Cdh11 may be involved in the regulation of PC maturation in ASD-relevant areas.

### Delayed dendritic development of Purkinje cells in Cdh11-exprssing area

**Fig. 6:**
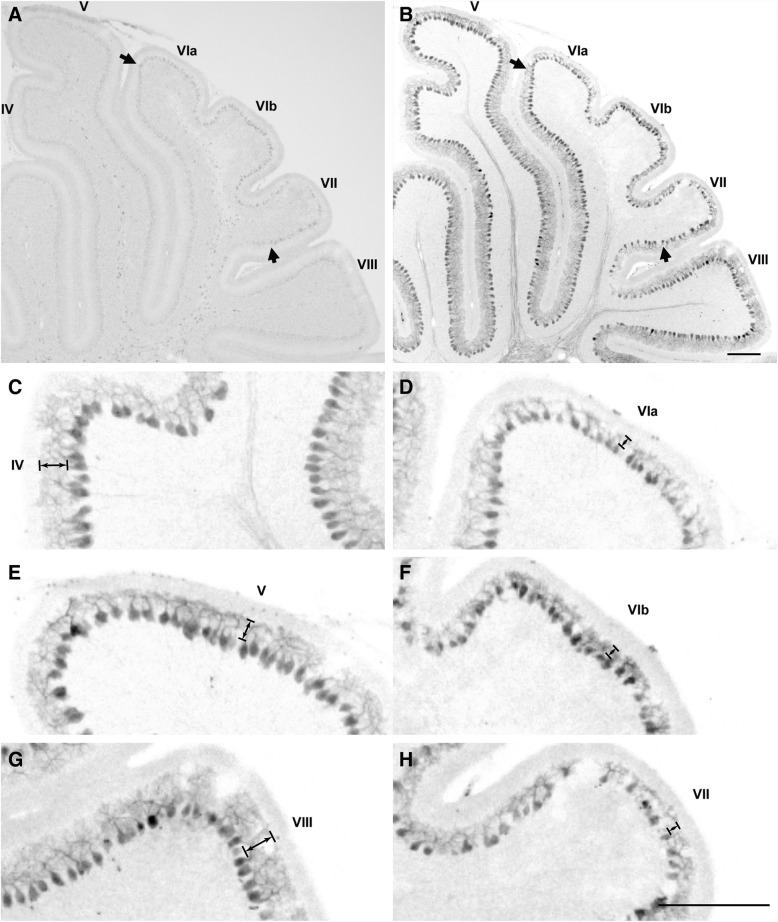
Delayed dendritic development of Purkinje cells in lobules VI/VII at P10. In situ hybridization followed by calbindin staining was performed on P10 sagittal sections. **a**, **b** shows an example sagittal section from the vermis. **a** In situ hybridization signals of Cdh11. Arrows mark the central area of lobules VI/VII with Cdh11 mRNA expression. **b** Counterstaining of calbindin on the same section shows the general morphology of Purkinje cells. Arrows mark the same area of lobules VI/VII in (**a**). **c**, **e** and **g** are enlarged images of parts of lobules IV, V, and VIII, respectively, from (**b**). **d**, **f** and **h** are enlarged images of parts of lobules VIa, VIb, and VII, respectively, from (**b**). Arrows indicate that lobules VIa, VIb, and VII have a much thinner molecular layer than that of lobules IV, V, and VIII. Scale bars: 200 μm

Vermal lobules VI/VII are known to be the latest developed lobules of the CB [46]. After the first postnatal week (P10), Cdh11 expression in PCs in the central region of vermal lobules VI/VII was found to be high (Additional file 2: Figure S2), and the molecular layer of this region was much thinner than that of nearby lobules (Fig. 6). In flanking regions with a high Cdh9 expression level, the thickness of the molecular layer was comparable with that of other lobules (Fig. 6). These data further support the notion that Cdh11 expression correlates with a delayed maturation of PCs in vermal lobules VI/VII.

### Expressions of Cdh9 and Cdh11 in inferior olivary nucleus

**Fig. 7:**
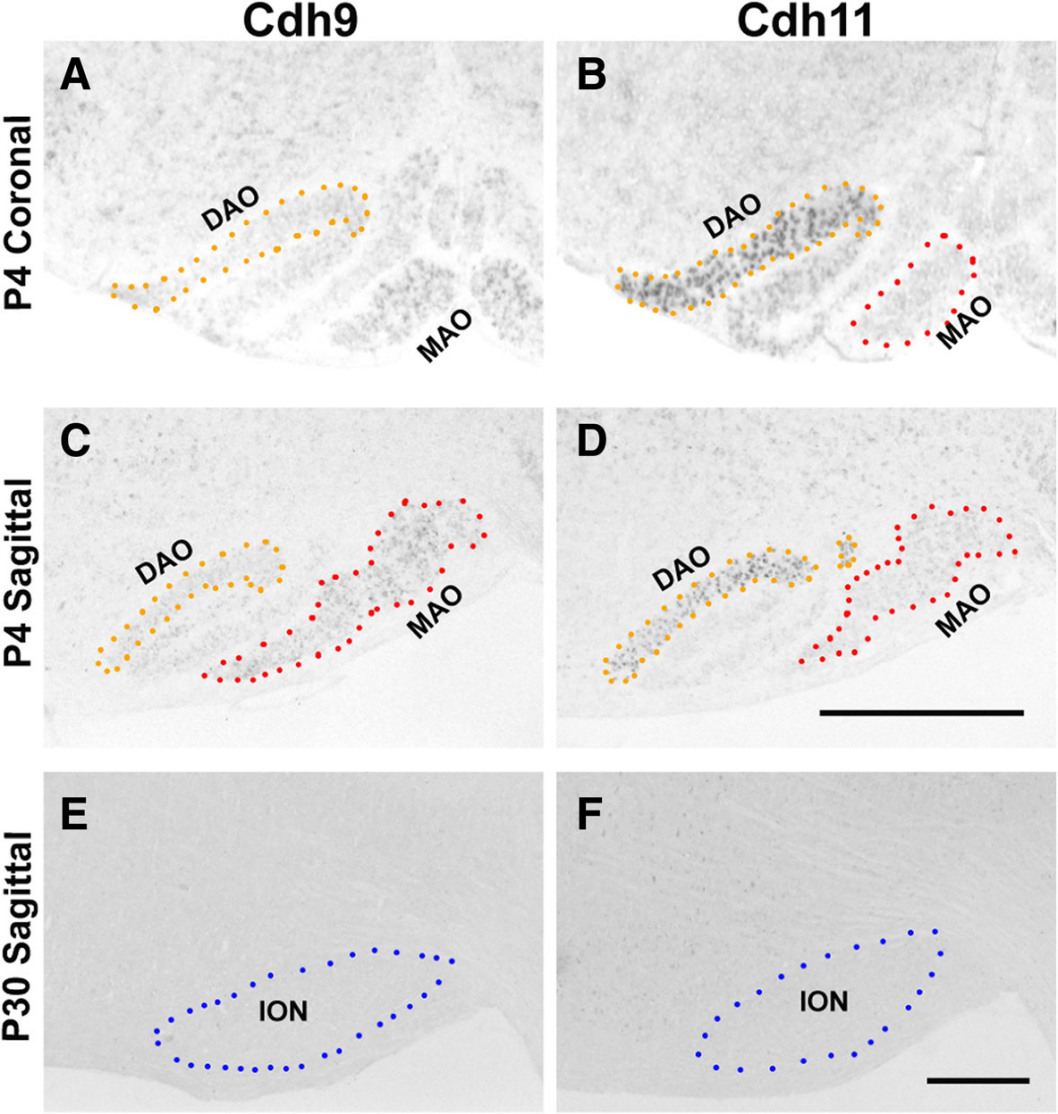
Expressions of cadherins in inferior olivary nucleus (ION). In situ hybridization was performed on coronal and sagittal sections at different stages. **a** and **b** sections show Cdh9 and Cdh11 expression on two adjacent coronal sections at P4, suggesting that they are expressed in different olivary sub-nuclei. Cdh11 is expressed in dorsal accessory olivary nucleus (DAO), whereas Cdh9 is expressed in medial accessory olivary nucleus (MAO). **C** and **D** sections show Cdh9 and Cdh11 expression on two adjacent sagittal sections at P4, further indicating their expression in different sub-nuclei. **e**, **f** Cdh9 and Cdh11 expression is absent in ION at P30. Scale bars: 500 μm

Neurons of the inferior olivary nucleus (ION) send climbing fiber axons to make synaptic connections with PCs. The segregated expression of Cdh9 and Cdh11 in the PC layer of the CB raised the question as to whether their presynaptic counterparts also express the two ASD-associated cadherins in a segregated pattern. By in situ hybridization, we found that Cdh11 was highly expressed in the dorsal accessory olivary nucleus (DAO), whereas Cdh9 was weakly expressed in the medial accessory olivary nucleus (MAO) (Fig. 7). These data suggest that the two ASD-associated genes are expressed in distinct sub-nuclei of ION.

## Discussion

The CB has a highly compartmentalized organization in anatomy and function. At the medial-lateral dimension, the mature CB includes three anatomical subregions: the vermis (medial CB), the paravermis (intermediate CB or pars intermedia), and the hemisphere (lateral CB). Each of these subregions are folded into different lobules at the anterior-posterior dimension [47]. The lobulation of the CB is controlled by genetic factors and is largely conserved across species [48]. Each lobule is further organized into a series of parasagittal stripes (bands) that are defined by the expression of certain molecular markers [49–51]. Different parts of the CB are functionally coupled to specific neural systems. Anterior lobules I–V and lobule VIII are mainly involved in sensorimotor pathways, and posterior lobules VI-VII/Crus I–II play important roles in cognitive functions [52]. Lobules VI-VII/Crus I–II are also regions of the CB that are closely associated with ASD, and have been shown to connect anatomically or functionally with brain regions that play important roles in language, cognition, and emotion. For example, trans-synaptic viral tracing and electrophysiological mapping in monkeys and rodents have revealed a bidirectional loop between the dorsolateral prefrontal cortex and the lateral crus II and the vermal lobule VII [53]. Resting-state functional connectivity MRI demonstrated that lobules VI/VII of the vermis are very likely to be associated with mid-frontal regions including the anterior cingulate cortex in humans [54], and retrograde trans-neuronal tracing using rabies virus showed connection of homologous regions with lobules VII in rats [55]. Lobules VI/VII are also a prominent cerebellar visual-receiving area [56, 57], which may be relevant to the oculomotor deficits observed in individuals with ASD. In the present study, we observed a high level of expression of Cdh9 and Cdh11 in the PC layer of lobules VI-VII/Crus I–II, supporting the notion that these two newly identified ASD risk genes may play important roles in the development and function of PCs in lobules VI-VII/Crus I–II. It would be interesting to investigate whether PCs of lobules VI-VII/Crus I–II exhibit anatomical and physiological alterations in Cdh9 or Cdh11 knockout mice and whether these mutant mice display behavioral deficits relevant to the known functions of lobules VI-VII/Crus I-II in ASD, e.g., impaired sociability and increased repetitive behavior [7, 14, 58].

Two studies using Cdh11 KO mice reported that these mutant mice exhibit low acoustic startle response [59, 60], which could be partly attributed to a moderate hearing loss due to abnormal development of the middle ear [60]. These mutant mice have reduced prepulse inhibition (PPI) amplitude in the acoustic startle response at an 80-dB prepulse level and reduced startle response induced by air-puff, but not by electrical foot-shocks [60]. These behavioral abnormalities may not involve Cdh11 in lobules VI-VII/Crus I-II of the CB, since PC circuitries do not seem to be essential for PPI of acoustic startle response [61, 62]. Cdh11 KO mice show reduced anxiety levels [59] and enhanced context-dependent freezing [60]. These behavioral abnormalities may be attributed to impaired function of the limbic system, particularly the amygdala and hippocampus, which have high levels of Cdh11 expression during the developmental stage [59]. Whether these mutant animals have increased repetitive behavior remains to be investigated.

Involvement of a subarea of the CB in specific physiological functions depends largely on afferent and efferent connections [63–65]. One major afferent afferent to PCs are climbing fibers emanating from the ION. The topographic wiring between climbing fibers and PCs during development is determined by chemospecific interactions of cell surface molecules [63]. Several guidance molecules, including Eph receptors and Ephrins ligands, have been shown to play important roles in the establishment of stripe-specific connections between climbing fibers and PCs [64, 65]. However, guidance molecules that mediate the wiring of climbing fibers to specific lobules remain largely unknown. Type II cadherins have been suggested to play an instructive role in mediating axon targeting and synaptic specificity through homophilic interactions [66]. We found that Cdh9 and Cdh11 are expressed in two distinct subgroups of PCs. Since Cdh9 and Cdh11 are also expressed in ION, which sends out olivocerebellar climbing fibers to innervate PCs, they may affect the synaptic specificity of two different sub-groups of PCs with afferent climbing fibers. Is it likely that Cdh9-expressing PCs and Cdh11-expressing PCs receive synaptic connections from part(s) of the ION with the expression of the same type of cadherins? We found that Cdh11 expression in ION was largely restricted to DAO and that Cdh9 was expressed mainly in MAO. However, based on results of previous axon tracing studies [67], neither the vermal lobules VI/VII nor the lateral hemisphere receives climbing fibers from DAO, and the lobule VI/VII region is only very sparsely innervated by MAO. Therefore, it seems unlikely that homophilic interactions of Cdh11 and Cdh9 play a major role in topographic innervation between climbing fibers and PCs. During the development of the retina, a heterophilic interaction between Cdh9 and Cdh8 has been shown to contribute to the establishment of synapse specificity of a direction-selective retinal circuit [68]. It remains to be determined which cadherin family members interact with Cdh9 or Cdh11 to regulate the wiring of olivocerebellar circuits via a heterophilic mechanism. However, an important role of homophilic interactions of other cadherin family members in the determination of synaptic specificity of the olivocerebellar circuit cannot be ruled out.

In addition to Cdh9 and Cdh11, previous studies showed that several other type II cadherins, including Cdh6, and Cdh8, are also differentially expressed in cerebellar lobules and ION [37, 40]. Our finding of DAO-specific expression of Cdh11 is consistent with the results of these previous studies. Several genetic studies have implicated Cdh8 as another ASD susceptibility gene [25, 69]. In neonatal mice, Cdh8 exhibits a broad expression in ION. In addition to MAO and DAO, Cdh8 is also expressed in the principal olive (PO) [37, 40], which sends climbing fibers to innervate the hemisphere of the CB [67, 70]. Interestingly, the expressions of Cdh11 and Cdh8 largely overlap in the hemisphere [40], one of the regions of the CB that are closely associated with ASD. An intriguing possibility is that Cdh8 may mediate specific innervation of the cerebellar hemisphere by climbing fibers emanating from the PO through a homophilic interaction of Cdh8 between climbing fibers and PCs and a heterophilic interaction between Cdh8 in climbing fibers and Cdh11 in PCs. The broad expression of Cdh8 in major sub-nuclei of ION also suggests that it may regulate the projection of climbing fibers through heterophilic interactions with multiple cadherin family members that are expressed in different lobules of the CB.

In the developing CB of both chicken and mouse, Cdh11 is also expressed in some granule cell raphes, a transient structure composed of migratory granule cells between PC clusters at intermediate stages of development [37, 71], suggesting that Cdh11 may play a role in the regulation of granule cell migration at this stage. Unlike Cdh6, Cdh7, and Cdh8, which are expressed in both PCs and subregions of deep cerebellar nuclei, Cdh11 expression in deep nuclear region is very low [37], suggesting that it may not play a major role in the topographic connectivity between PCs and the deep cerebellar nuclei.

Lobules VI/VII belong to the latest developing cerebellar structures. Delayed maturation of lobule VI/VII PCs compared to that of anterior and posterior lobules has been consistently observed in different species [46, 72, 73]; this may be a reason for its frequent involvement in developmentally related disturbances and disorders. Based on our observations, Cdh11-expressing region of lobule VI/VII displays delayed maturation, as reflected by a much lower expression of calbindin-D28k and a much thinner molecular layer than that of Cdh9-expression regions in neighboring lobules at P7 and P10 (Figs. 5 and 6). We noted that at P4 and P7, calbindin expression signal in the central region of lobules VI/VII was very low, most likely dues to the delayed maturation of PCs in this region. Consequently, calbindin immuno-staining could indicate the PC layer but could not display the clear morphology of individual PCs in this region, unlike the clear labeling of PC morphology in neighboring lobules in the same brain section. At this stage, we could see strong Cdh11 expression signal within the calbindin-positive cell layer of the central region of lobules VI/VII, most likely in very immature PCs. At P10, however, with further maturation of PCs, calbindin expression level becomes much higher, and the expression of Cdh11 in calbindin-positive PCs in this region can be better visualized (Additional file 2: Figure S2). Altogether, these observations support our notion that Cdh11 expression in PCs of the central region of lobule VI/VII correlates with a delayed maturation of these PCs.

Neurons rich in calcium-binding proteins, especially calbindin-D28k and parvalbumin, appear to be relatively resistant to degeneration in a variety of acute and chronic disorders [74]. This indicates that Cdh11-expressing PCs in ASD-associated areas during early postnatal development may be more vulnerable to cell-damaging factors due to its low expression of calbindin. Whether there is a causal link between Cdh11 high expression and a delayed maturation of PCs remains to be clarified.

In the developing CB, pruning of climbing fiber occurs after the first postnatal week; this is a process essential to the establishment of topographic wiring of PCs [75, 76]. It has been postulated that incomplete synaptic pruning is a underlying cellular mechanism for ASD [77]. Previous findings suggest that a high expression of Cdh11 may increase the synaptic density in cultured neurons [78–80]. During normal PC development, a properly timed developmental pruning of synaptic inputs occurs to ensure the completion of neuronal maturation [81]. An intriguing possibility is that the initial high expression of Cdh11 may render sufficient number of synaptic contacts from climbing fibers onto PCs in ASD-associated areas, whereas a subsequent downregulation of Cdh11 by the end of the first postnatal week may trigger the pruning process. Future studies in Cdh11 knockout mice are required to address whether malfunction of Cdh11 indeed perturbs the synaptic pruning process of PCs, thereby disrupting normal cerebellar motor and/or cognitive functions.

The segregated expression patterns of Cdh9 and Cdh11 implies that the two genes may contribute to the determination of sub-regions of autism-associated areas in the CB. A well-known example of tissue compartmentalization by genes of the same family is the animal body patterning by a subset of homeobox-containing transcription factors known as Hox genes. During development, spatial-temporally coordinated expression of distinct Hox family members determines the boundaries of different body segments [82]. The loss-of-function of one Hox gene usually results in the expansion of another gene’s territory. Interestingly, several Hox family members, including HoxA1 and HoxB1, have been suggested to be associated with ASD [83–86], and mutations of HoxA1 has been shown to affect CB morphogenesis [87]. It would be interesting to use gene knockout mice to examine whether Cdh9 and Cdh11 are downstream targets of transcription factors of the Hox family and, whether loss-of-function of one cadherin leads to compensatory expansion of the area expressing the other.

## Additional files

Additional file 1: **Figure S1.** Comparison of Cdh9 and Cdh11 anti-sense and sense probes for in situ hybridization. In situ hybridization using sense and antisense probes for Cdh9 and Cdh11 was performed on two adjacent sagittal sections at P0. A and B sections show that hybridization signal was detected on the section with Cdh9 antisense probe but not with Cdh9 sense probe in the corresponding areas (arrows). C and D sections show that hybridization signal was detected on the section with Cdh11 antisense probe but not with Cdh11 sense probe in the corresponding areas (arrows). Scale bar: 500 μm. (TIF 9092 kb)

Additional file 2: **Figure S2.** Expression of Cdh11 in Purkinje cells in lobules VI/VII at P10. In situ *hybridization* followed by calbindin staining was performed on P10 sagittal sections. (A-D) Colocalization of Cdh11 in situ hibridization signal (green, pseudo color) with calbindin (red) in the vermis is demonstrated. (D) The enlarged image of the boxed area from (C) shows the co-localization of Cdh11 signal with calbindin in lobules VI/VII of the vermis. Scale bars: 500 μm. (TIF 8706 kb)

## Abbreviations

ASD: Autism spectrum disorder
CB: Cerebellum
Cdh11: Cadherin-11
Cdh9: Cadherin-9
DAO: Dorsal accessory olivary nucleus
DIG: Digoxigenin
ION: Inferior olivary nucleus
MAO: Medial accessory olivary nucleus
PC: Purkinje cell
