# Supplementary figures and images for: Segregated expressions of autism risk genes Cdh11 and Cdh9 in autism-relevant regions of developing cerebellum
Source: Mol Brain. 2019 May 2;12:40. doi: 10.1186/s13041-019-0461-4 (PMC6498582; doi:10.1186/s13041-019-0461-4)

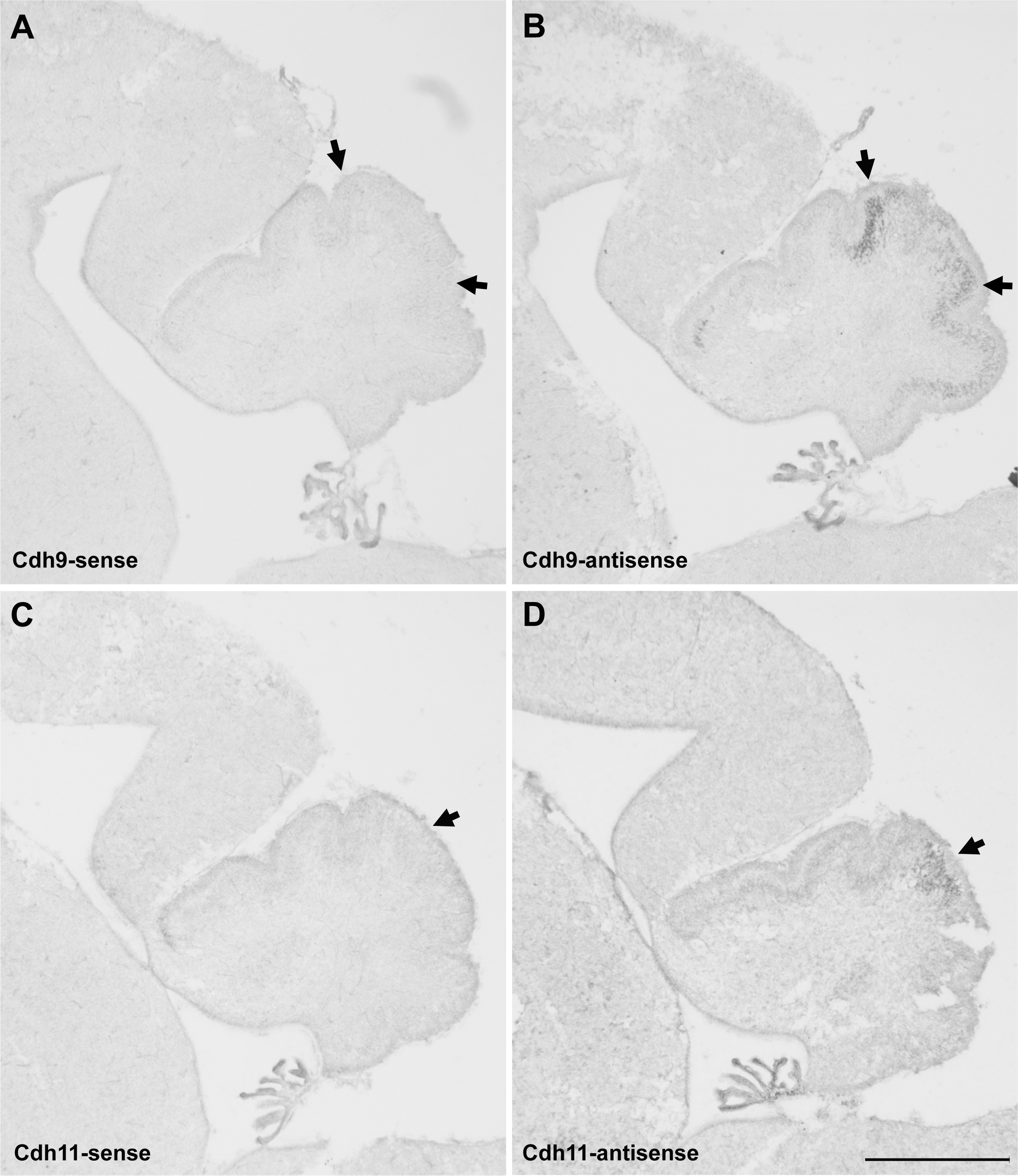

Supplement: Supplementary file 1 — Figure S1. Comparison of Cdh9 and Cdh11 anti-sense and sense probes for in situ hybridization. In situ hybridization using sense and antisense probes for Cdh9 and Cdh11 was performed on two adjacent sagittal sections at P0. A and B sections show that hybridization signal was detected on the section with Cdh9 antisense probe but not with Cdh9 sense probe in the corresponding areas (arrows). C and D sections show that hybridization signal was detected on the section with Cdh11 antisense probe but not with Cdh11 sense probe in the corresponding areas (arrows). Scale bar: 500 μm. (TIF 9092 kb) [file 13041_2019_461_MOESM1_ESM.tif]

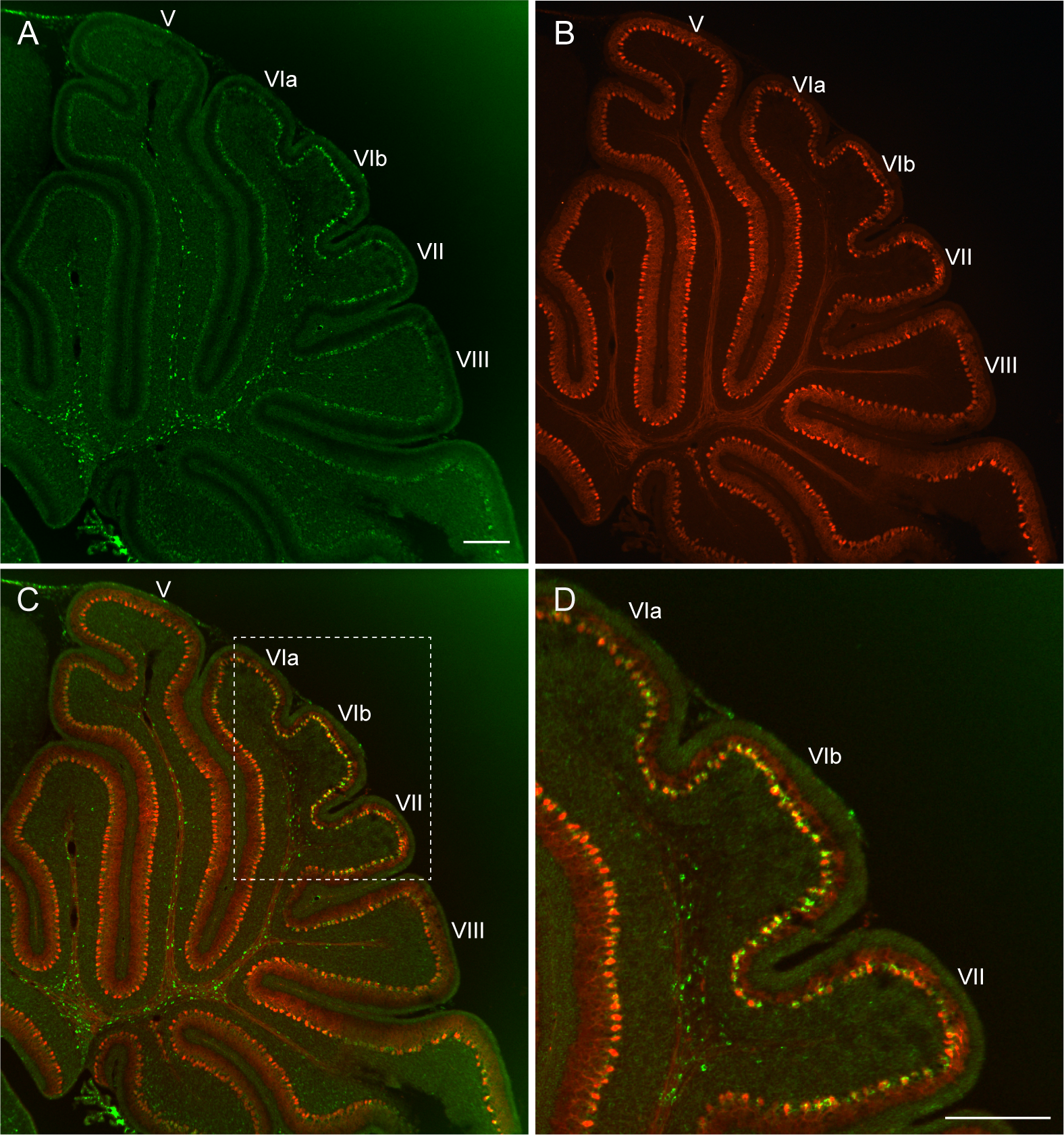

Supplement: Supplementary file 2 — Figure S2. Expression of Cdh11 in Purkinje cells in lobules VI/VII at P10. In situ hybridization followed by calbindin staining was performed on P10 sagittal sections. (A-D) Colocalization of Cdh11 in situ hibridization signal (green, pseudo color) with calbindin (red) in the vermis is demonstrated. (D) The enlarged image of the boxed area from (C) shows the co-localization of Cdh11 signal with calbindin in lobules VI/VII of the vermis. Scale bars: 500 μm. (TIF 8706 kb) [file 13041_2019_461_MOESM2_ESM.tif]
